# Social behaviours by *Bacillus subtilis*: quorum sensing, kin discrimination and beyond

**DOI:** 10.1111/mmi.14127

**Published:** 2018-11-01

**Authors:** Margarita Kalamara, Mihael Spacapan, Ines Mandic‐Mulec, Nicola R. Stanley‐Wall

**Affiliations:** ^1^ Division of Molecular Microbiology, School of Life Sciences University of Dundee Dundee DD15EH UK; ^2^ Department of Food Science and Technology, Biotechnical Faculty University of Ljubljana Ljubljana 1000 Slovenia

## Abstract

Here, we review the multiple mechanisms that the Gram‐positive bacterium *Bacillus subtilis* uses to allow it to communicate between cells and establish community structures. The modes of action that are used are highly varied and include routes that sense pheromone levels during quorum sensing and control gene regulation, the intimate coupling of cells *via* nanotubes to share cytoplasmic contents, and long‐range electrical signalling to couple metabolic processes both within and between biofilms. We explore the ability of *B. subtilis* to detect ‘kin’ (and ‘cheater cells’) by looking at the mechanisms used to potentially ensure beneficial sharing (or limit exploitation) of extracellular ‘public goods’. Finally, reflecting on the array of methods that a single bacterium has at its disposal to ensure maximal benefit for its progeny, we highlight that a large future challenge will be integrating how these systems interact in mixed‐species communities.

## Introduction

Although prokaryotes are widely viewed as single‐celled organisms, many forms of multicellularity are prevalent in the bacterial world. Bacterial multicellularity can be transient or permanent; for example, cells of some species can form aggregates and filaments temporarily, while others, such as filamentous Cyanobacteria, form permanent chains of differentiated cells (Claessen et al., 2014). Multicellular lifestyles have evolved independently in different bacterial species and are characterised by cell‐cell adhesion, division of labour, and intercellular cooperation (Claessen et al., 2014; Lyons and Kolter, 2015). Communal living provides bacteria with a multitude of benefits: resistance to environmental threats, increased nutrient acquisition, protection from predation and more efficient utilisation of available resources through cell differentiation (Lyons and Kolter, 2015). Intercellular cooperation is often mediated by the production of ‘public goods’, which are molecules that are produced by a subpopulation of cells in a community but are shared with producers and non‐producers alike (West et al., 2006). As public goods are secreted, extracellular products, they are also susceptible to exploitation by ‘cheaters’; cells that take advantage of the molecules produced by their neighbours without directly contributing to their production (Rainey and Rainey, 2003; Diggle et al., 2007; Sandoz et al., 2007; West et al., 2007). Given this, bacteria need not only to discriminate between species that are beneficial to cooperate with, and those that need to be competed against but also need to make similar decisions about isolates of the same species. A mechanism by which this process occurs is ‘kin discrimination’; the differential treatment of organisms based on how closely related they are. In such systems, conspecific cells (cells of organisms belonging to the same species) that are recognised as self are cooperated with, while cells that are recognised as non‐self are competed against [as reviewed by (Hamilton, 1964; Strassmann et al., 2011; Wall, 2016)]. Here, we review the recent advances in understanding the social interactions between isolates of the Gram‐positive bacterium *Bacillus subtilis* highlighting the diversity of communication mechanisms that have evolved, while exploring their links with establishing a social, community life in a biofilm.

## Multicellularity in *Bacillus subtilis*

**Figure 1:**
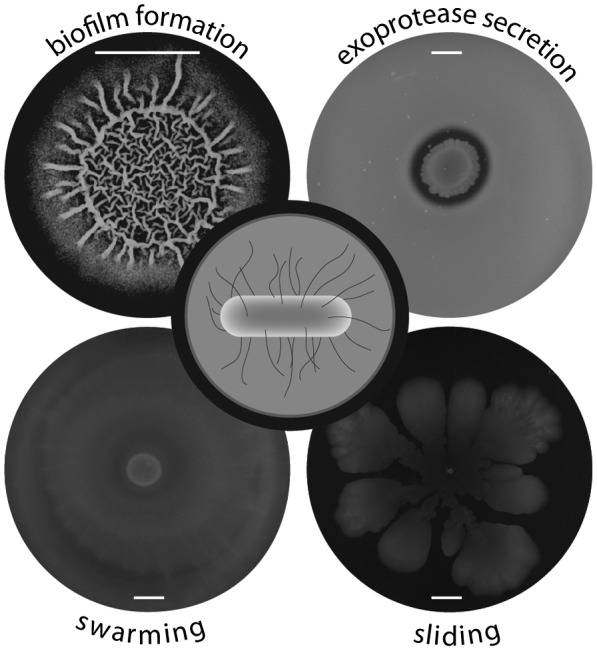
Multicellular behaviours exhibited by *B. subtilis*. Biofilm formation, assessed after growth on MSgg agar and imaged 48 h after growth at 30°C (top left) [method from (Branda et al., 2001)]. Protease secretion tested on LB+ 1% milk (w/v) agar plates. The image was taken 18 h after growth at 37°C (top right) [method from (Verhamme et al., 2007)]. Swarming motility assessed on low salt LB agar + 0.7% agar (w/v) plates and imaged 8 h after incubation at 37°C (bottom left) [method from (Kearns and Losick, 2003)]. Sliding motility tested by growth on MsggN plates for 72 h at 37°C (bottom right) [method from (Fall et al., 2006)]. In each case, the *B. subtilis *undomesticated isolate NCIB 3610 was used.

*Bacillus subtilis* is a soil organism that exhibits a multitude of social (multicellular) behaviours including swarming (Kearns and Losick, 2003) and sliding motility (Kinsinger et al., 2003), exoprotease production (Wu et al., 1991; Dahl et al., 1992; Msadek, 1999) and biofilm formation (Branda et al., 2001; Hamon and Lazazzera, 2001) (Fig. 1). Swarming and sliding motility allow bacteria to colonise nutrient rich environments through flagella‐dependent and flagella‐independent processes respectively (Henrichsen, 1972; Fraser and Hughes, 1999). Each of these motility mechanisms, and biofilm formation (Branda et al., 2001), depends on the production of surfactin, a secreted lipopeptide that lowers surface tension allowing movement of the cells over a surface (Kearns and Losick, 2003; Kinsinger et al., 2003; Kinsinger et al., 2005). Exoprotease production facilitates the breakdown of complex molecules, allowing access to nutrients (Msadek, 1999) and biofilm formation is mediated by the production of the biofilm matrix which provides the community with stability and protection (Flemming and Wingender, 2010). Due to the gene regulatory networks controlling their synthesis, it is likely that the production of many of the molecules that act as public goods are stimulated when *B. subtilis* reaches high density, through a process of quorum sensing.

## Quorum sensing in *B. subtilis*

Quorum sensing (QS) is a cell‐cell communication mechanism that allows bacteria to coordinate physiological processes in response to cell density (Miller and Bassler, 2001; Henke and Bassler, 2004). Bacteria secrete signals called autoinducers into the extracellular environment and, as the concentration of autoinducers increases, this stimulates activation of downstream gene expression (Miller and Bassler, 2001; Henke and Bassler, 2004). Besides being a density‐dependent mechanism QS has also been indicated as a diffusion sensing (Redfield, 2002) and/or efficiency sensing mechanism (Hense et al., 2007). In *B. subtilis*, QS systems both directly and indirectly control public good production (Oslizlo et al., 2014; Spacapan et al., 2018) and cooperative behaviours (Schuster et al., 2013). To date there have been no quorum sensing deficient isolates of *B. subtilis *isolated, a finding that is consistent with cooperative behaviours being crucial for survival.

**Figure 2:**
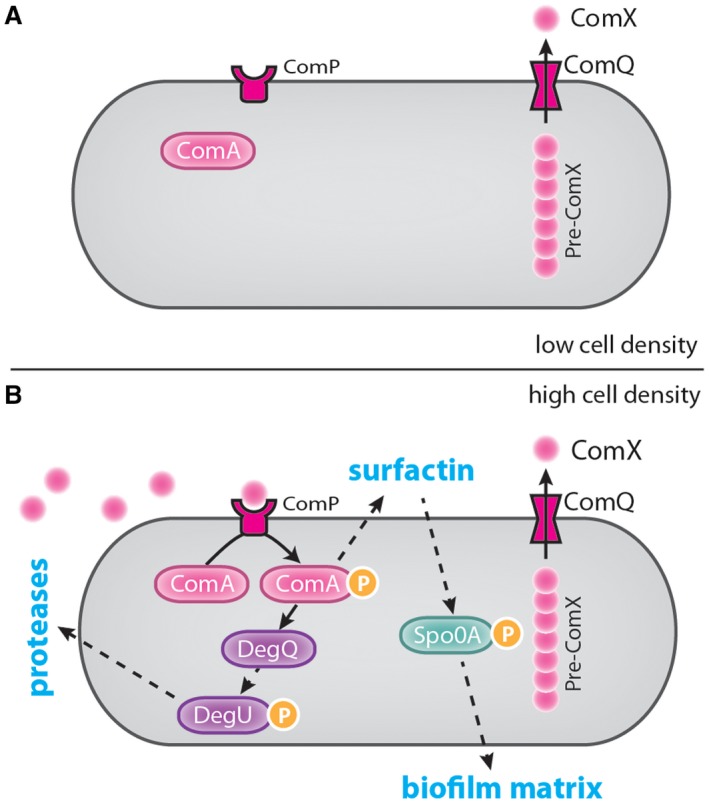
The ComXQPA quorum sensing system in *Bacillus subtilis*. Illustration of the function of the ComXQPA system in *B. subtilis *under low (A) and high (B) cell density conditions. Dashed arrows represent indirect regulation. Pre‐ComX (chain of circles) is synthesised in the cell, modified and exported by ComQ, resulting in secretion of the ComX pheromone (single circle). ComP is the ComX receptor. In low cell density conditions, the extracellular concentration of ComX is low and ComP does not bind ComX. Under high cell density conditions, however, the extracellular concentration of ComX increases and ComX binds ComP. ComP phosphorylates and activates ComA. ComA~P subsequently facilitates surfactin production and activates the production of DegQ. DegQ indirectly controls the phosphorylation and activation of DegU, leading to production and secretion of exoproteases. Secretion of surfactin indirectly causes phosphorylation of Spo0A and Spo0A~P facilitates production of the extracellular matrix.

A well‐studied QS system in *B. subtilis* comprises the proteins ComQXPA. ComX is the autoinducer (pheromone) (Magnuson et al., 1994) and ComP is the sensor protein kinase (Weinrauch et al., 1990; Piazza et al., 1999) that is part of the ComP‐ComA two‐component signal transduction system, with its cognate DNA‐binding response regulator ComA (Roggiani and Dubnau, 1993; Wolf et al., 2016). ComQ is required for processing, modification and export of ComX and consequentially production of the mature QS signal (Ansaldi et al., 2002; Bacon Schneider et al., 2002). Extracytoplasmic binding of ComX to the receiver domain of ComP leads to phosphorylation and activation of ComA in the cytoplasm (Roggiani and Dubnau, 1993). It is the phosphorylated form of ComA that positively regulates production of surfactin (Nakano et al., 1991b), and indirectly activates the production of other public goods (Comella and Grossman, 2005; Lopez et al., 2009a) through regulation of *degQ* transcription (Msadek et al., 1991; Spacapan et al., 2018). DegQ modulates DegU phosphorylation and consequently influences synthesis of exoproteases and other extracellular enzymes (Kobayashi, 2007) (Fig. 2).

Among isolates of *B. subtilis *the locus encoding the ComQXPA system is highly polymorphic (Tran et al., 2000; Tortosa et al., 2001; Ansaldi et al., 2002; Stefanic and Mandic‐Mulec, 2009; Oslizlo et al. 2015). More specifically, the coding regions for *comQ*, *comX* and 5′ end of *comP* are poorly conserved, leading to divergence of isolates into separate social communication groups or ‘pherotypes’ (Tran et al., 2000; Tortosa et al., 2001; Ansaldi et al., 2002; Stefanic and Mandic‐Mulec, 2009; Oslizlo et al., 2015). What drives evolution of the polymorphisms in this QS system is currently unknown but the diversity generated allows *B. subtilis* to be categorised into distinct pherotypes that fail to ‘listen’ and respond to each other. Striking diversity of pherotypes that use distinct languages for communication is evident even among isolates found in a single cm^3^ of soil or on root surfaces of a single plant (Stefanic and Mandic‐Mulec, 2009; Oslizlo et al., 2015). The diversity in the quorum sensing groups strongly correlates with the phylogenetic and ecological relationship among *B. subtilis* isolates, such that closely related isolates that belong to the same ecological group, typically also share a pherotype (Stefanic et al., 2012). Thus, *B. subtilis* primarily communicates with other isolates of its own ecologically distinct group or ‘ecotype’. Despite this, there are exceptions to the rule as there are usually minority pherotypes within an ecotype, which can communicate with isolates of different ecotypes in the local environment (Stefanic et al., 2012).

Two models have been proposed for the coexistence of diverse pherotypes in the natural *B. subtilis *isolates. First, the observed diversification of the quorum sensing alleles may be a result of the ecotype diversity (Stefanic et al., 2012). Different ecotypes may vary in the environmental conditions where QS is used and therefore the sharing of QS signals between distinct ecotypes and the resulting public goods would be problematic. The second model proposed is the ‘pherotype cycling model’, in which a minority pherotype in a population would have an advantage by exploiting the communal goods produced by other cells in the population (Stefanic et al., 2012). The minority pherotype in the community only has this advantage when below the threshold level for quorum sensing and public good production. It is predicted that ‘cheaters’ would increase in abundance with time, due to their fitness advantage, and would eventually be the predominant pherotype in the population. Then at this stage the previously dominant pherotype would become the cheater and so on (Stefanic et al., 2012).

## Quorum sensing and facultative cheating

Cheating and pherotype diversity in *B. subtilis* have been explored by a theoretical model (Eldar, 2011) and further analysed using an experimental system in which the *comQXP* locus of strains belonging to four different pherotypes were introduced individually, such that the only difference in the otherwise isogenic strains was the *comQXPA* QS allele (Pollak et al., 2016). Co‐culturing various pairs of strains in a swarming co‐culture assay uncovered that in almost all cases the minority pherotype had a fitness advantage over the majority (Pollak et al., 2016). These findings are consistent with the previously proposed pherotype cycling model (Stefanic et al., 2012). It is worth noting that there were a few exceptions to the rule, most likely due to asymmetric signalling, where the autoinducer of one pherotype interferes with the signalling of the other (Pollak et al., 2016), as has been previously demonstrated in liquid cultures of *B. subtilis* (Ansaldi et al., 2002).

**Figure 3:**
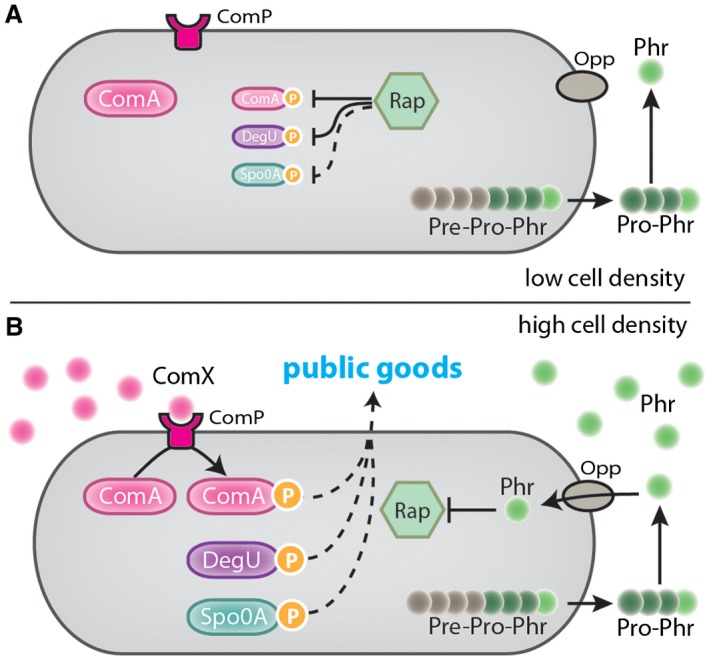
The ComXQPA and Rap/Phr quorum sensing systems in *Bacillus subtilis*. Schematic of the quorum sensing systems under low (A) and high (B) cell density conditions. Pre‐Pro‐Phr is synthesised in the cytoplasm. The signal peptide (represented in brown circles) is cleaved off and Pro‐Phr is secreted and modified in the extracellular environment to produce the Phr peptide (light green circle). Under low cell density conditions, the extracellular concentrations of Phr are low and Phr does not enter the cell. The Rap protein represses the response regulators ComA~P, DegU~P and indirectly Spo0A~P. Under high cell density conditions, the extracellular concentration of Phr increases and the Phr peptide enter the cells through the Opp system. Phr represses Rap allowing ComA~P, DegU~P and Spo0A~P to facilitate the production of public goods.

While the ComQXPA system is important for determining the social communication group that an isolate belongs to, it is not the only QS system as *B. subtilis* also utilises the Rap‐Phr QS systems (Perego and Hoch, 1996; Lazazzera et al., 1997). In contrast to ComQXPA, where there is a single system encoded by the genome, each *B. subtilis* isolate encodes multiple Rap‐Phr systems with considerable strain specificity in the Rap‐Phr pairs encoded being evident (Even‐Tov et al., 2016b) (Table 1). For example, the genome of *B. subtilis* 168 encodes eight receptor‐signal pairs of the Rap‐Phr system (namely, Rap‐Phr A, C, E, F, G, H, I, K) as well as three orphan receptors (namely, RapB, D and J) (Kunst et al., 1997; Jiang et al., 2000; Omer Bendori et al., 2015). Interestingly, the QS systems in *B. subtilis* appear to converge and regulate the same physiological responses (Even‐Tov et al., 2016b) (Table 1). This is accomplished as several different Rap proteins repress activity of ComA, the response regulator of the ComQXPA system (Solomon et al., 1996; Lazazzera et al., 1999; Auchtung et al., 2006; Ogura and Fujita, 2007). When cells are at a low cell density, the Rap proteins additionally control other regulators of public good production including DegU (Ogura et al., 2003; Hayashi et al., 2006) and Spo0F (Perego et al., 1994; Perego and Hoch, 1996; Perego, 1997; Jiang et al., 2000; Parashar et al., 2011; Rosch and Graumann, 2015). Spo0F is part of the Spo0A phosphorelay system (Burbulys et al., 1991) which ultimately controls expression of biofilm matrix genes through modulating the levels of phosphorylated Spo0A (Hamon and Lazazzera, 2001). DegU is involved in the regulation of cooperative processes such as genetic competence (Roggiani et al., 1990; Msadek et al., 1991), swarming motility (Amati et al., 2004), exoprotease secretion (Dahl et al., 1992) and biofilm formation (Stanley and Lazazzera, 2005; Verhamme et al., 2007). When the bacterial population reaches a quorum, the Phr peptides accumulate and repress the Rap proteins (Pottathil and Lazazzera, 2003), thereby allowing the response regulators to trigger expression of genomic regions involved in multicellular behaviours (Fig. 3). Thus, Rap systems firstly act antagonistically to the ComQXPA system as phosphatases or anti‐activators of ComA~P (Baker and Neiditch, 2011), but then, through the binding of the specific Phr peptides to their cognate Rap receptors, this inhibition is relieved (Parashar et al., 2013a; Even‐Tov et al., 2016b).

**Table 1:** Reported Rap‐Phr systems in *B. subtilis *isolates.

Rap Protein	Phr peptide	Location of cassette	Physiological function regulated	References
RapA	PhrA	Chromosome	Control of sporulation initiation; Dephosphorylates Spo0F	(Perego et al., 1994; Perego and Hoch, 1996)
RapB	‐ (PhrC inhibits RapB)	Chromosome	Control of sporulation initiation; Dephosphorylates Spo0F	(Perego et al., 1994; Perego, 1997)
	(Perego, 1997)			
RapC	PhrC	Chromosome	Control of ComA activity; Interacts with ComA and ComA~P	(Solomon et al., 1996; Lazazzera et al., 1999; Auchtung et al., 2006)
RapD	–	Chromosome	Inhibition of surfactin production; Control of ComA activity	(Ogura and Fujita, 2007)
RapE	PhrE	Chromosome	Control of sporulation initiation; Dephosphorylates Spo0F	(Jiang et al., 2000)
RapF	PhrF	Chromosome	Control of ComA activity; Interacts with ComA and ComA~P	(Bongiorni et al., 2005; Auchtung et al., 2006)
RapG	PhrG	Chromosome	Control of DegU; Interacts with DegU~P	(Ogura et al., 2003; Hayashi et al., 2006)
RapH	PhrH	Chromosome	Control of sporulation initiation and ComA activity; Dephosphorylates Spo0F	(Hayashi et al., 2006; Mirouze et al., 2011)
				(Parashar et al., 2011)
RapI	PhrI	Chromosome	Control of transfer of mobile genetic element ICE*Bs*1; Dephosphorylates Spo0F	(Rosch and Graumann, 2015)
			Crystal structure of RapI	(Parashar et al., 2013a)
RapJ	–	Chromosome	Control of Spo0A phosphorelay	(Parashar et al., 2011)
			Crystal structure of RapJ with CSF:	(Parashar et al., 2013a)
RapK	PhrK	Chromosome	Control of ComA activity	(Auchtung et al., 2006; Parashar et al., 2013a)
RapP	PhrP	pBS32	Control of biofilm formation (via modulation of ComA activity); PhrP does not counteract RapP due to a mutation in *rapP*.	(Parashar et al., 2013b)
RapQ	PhrQ	pBSG3	Control of sporulation, surfactin production and competency	(Yang et al., 2015)
Rap60	Phr60	pTA1060	Control of secreted protease production	(Koetje et al., 2003)
Rap_LS20_	Phr_LS20_	pLS20	Control of plasmid conjugation	(Singh et al., 2013; Rosch and Graumann, 2015)

**Figure 4:**
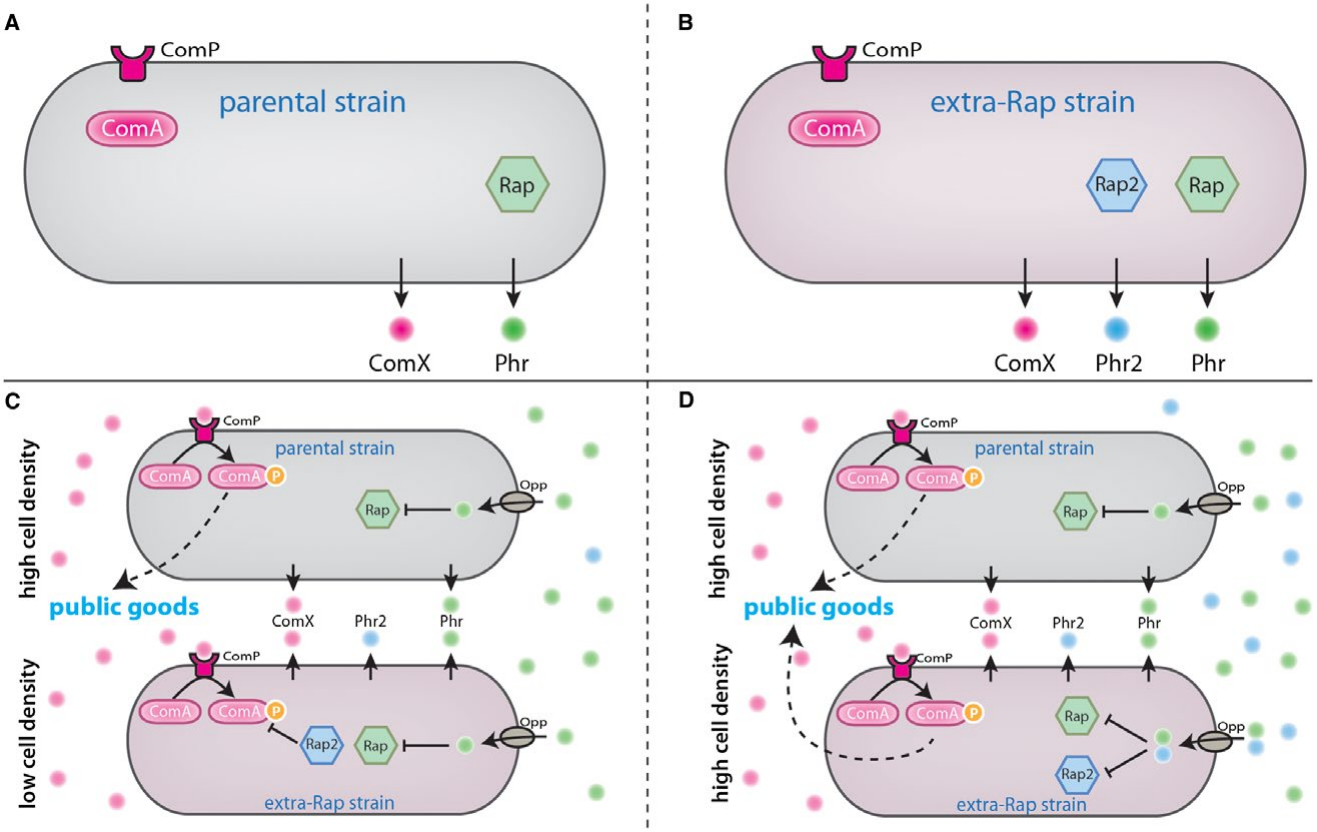
Quorum sensing and cheating. Schematic of the effect that acquisition of an additional Rap/Phr system has in *Bacillus subtilis *cheating. A. Representation of the parental strain, which encodes for the Com system and a single Rap/Phr system. The cell produces ComX pheromones and Phr peptides. B. The ‘Extra‐Rap’ strain, which has the same Com and Rap/Phr system as the parental strain plus an additional Rap/Phr (Rap2/Phr2, shown in blue) system. C. When the parental strain is at a quorum and the ‘Extra‐Rap’ strain is at a low density in the population, the extracellular concentrations of ComX and Phr are high, while Phr2 is present at low concentrations in the extracellular environment. ComX and Phr enter all cells (both the parental and ‘Extra‐Rap’). ComX leads to phosphorylation of ComA and Phr represses Rap. In the parental strain, ComA is free to facilitate public good production; while, in the ‘Extra‐Rap’ system, the absence of intracellular Phr2 results in a Rap2 repressing ComA~P, thereby repressing public good production. D. When both the parental strain and the ‘Extra‐Rap’ strain are at a quorum, public goods are produced by the parental strain as described in C). In the ‘Extra‐Rap’ strain, increased extracellular concentration of Phr2 results in the peptide entering the cell and repressing Rap2, allowing a contribution to public good production.

To test the evolutionary advantage of accumulating multiple Rap‐Phr systems in one strain, a combined experimental and mathematical modelling approach has been taken (Even‐Tov et al., 2016a). Introduction of an additional Rap‐Phr system (‘Extra‐Rap’ strain) was found to allow the exploitation of the parental strain, providing the derivative strain with a competitive advantage. This is due to antagonistic interactions between the QS systems. In the strain engineered to encode an additional Rap‐Phr system, when at low abundance, Rap proteins repress ComA leading to repression of public good production. The minority member of the population (the facultative cheater) containing the new quorum sensing system therefore avoids acting cooperatively and exploits the products secreted by the parental strain but becomes cooperative in the population when at a quorum (Even‐Tov et al., 2016a) (Fig. 4). Thus, two criteria have been proposed for a successful integration of a novel QS system into a strain: (1) the novel system must repress the ancestral QS system at low quorum and (2) addition of the autoinducer of the extra system must restore QS to a level similar to the ancestral strain (Even‐Tov et al., 2016a).

## Kin discrimination

**Figure 5:**
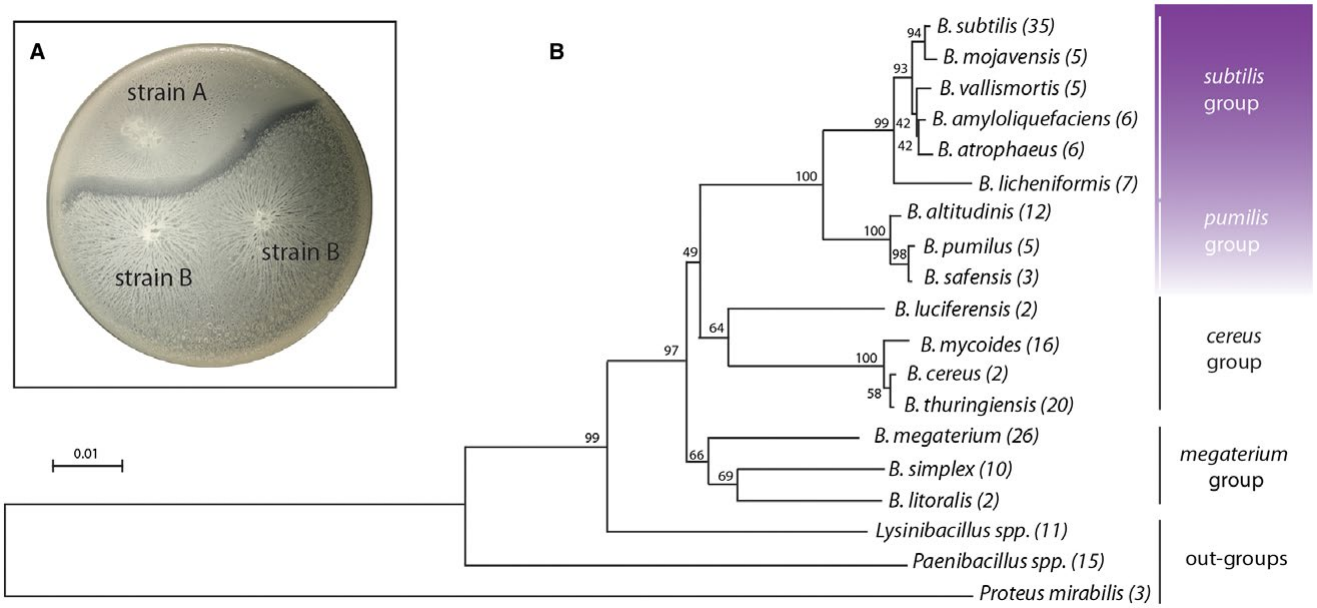
Kin discrimination in *B. subtilis*. A. Different phenotypes of approaching *B. subtilis* swarms can be used to distinguish kin and non‐kin strains of *B. subtilis.* Merging swarms indicate kin (two B strains) and a striking boundary indicates non‐kin swarms (strain A and strain B) (Stefanic et al., 2015); B. Phylogenetic tree adapted from Lyons and Kolter, 2017. The tree was calculated using the 16S rRNA sequence of a reference strain of each indicated species. The number of isolates of each species used in the study is indicated in parentheses. The separate clades are marked beside the tree and the purple gradient represents the cut‐off point for kin discrimination against *Bacillus subtilis *NCIB 3610.

It is postulated that to reduce exploitation of public goods by cheaters kin‐discrimination (KD) has evolved to stabilise cooperative behaviours among conspecific organisms that are recognised as ‘self’ (genetically identical individuals) or ‘kin’ (genetically related individuals of the same species that share cooperative genes and are able to cooperate) (Strassmann et al., 2011; Wall, 2016). Kin discrimination was identified in *B. subtilis *in 2015 by testing the ability of 39 natural isolates to cooperate by forming a common swarm (Stefanic et al., 2015) (Fig. 5A). Those isolates that were able to merge their swarms on agar surface were characterised as kin, while *B. subtilis* isolates that formed a visible boundary at the meeting point of the two swarms, were characterised as non‐kin (Stefanic et al., 2015). As discussed earlier, swarming motility is a cooperative behaviour, in which a group of cells migrates across a semi‐solid surface to acquire nutrients and requires the production of the public good surfactin (Kearns and Losick, 2003). Pairwise combinations of 39 isolates from two soil samples indicate that merging and thus cooperation only occurs in the most closely related strains where isolates with < 99.5% housekeeping gene identity (examined using the nucleotide sequences of *gyrA*, *rpoB*, *dnaJ* and *recA*) fail to recognise each other as kin. It was hypothesised that non‐kin form antagonistic rather than cooperative interactions (Stefanic et al., 2015). While there is a strong correlation between phylogenetic relationship, pherotype and kin recognition among isolates, the ability of strains to communicate with each other does not always equate to their ability to merge and potentially cooperate (Stefanic et al., 2015). Analysis of the 39 *B. subtilis* isolates showed that they belong to three different pherotypes (Stefanic and Mandic‐Mulec, 2009) and 12 kin recognition groups (Stefanic et al., 2015). These data suggest that not all isolates within the same pherotype can recognise each other as kin and, therefore, kin‐discrimination systems are likely to diversify faster than quorum sensing alleles and perhaps act as a mechanism to prevent cheating (Stefanic et al., 2015).

To test the effect that kin discrimination has on the formation of multicellular communities, *B. subtilis* strains that formed kin and non‐kin interactions during swarming were co‐inoculated onto plant roots. Consistent with *in vitro* studies, isolates belonging to the same kin recognition group formed mixed biofilms, while non‐kin strains engaged in antagonistic interactions resulting in one of the isolates primarily colonising the root (Stefanic et al., 2015). Next, the molecular factors involved in kin discrimination in *B. subtilis* NCIB 3610 and other *B. subtilis* strains such as FENS 2‐3‐5, COS39 and PS‐216 were uncovered by transposon mutagenesis and reverse genetics (Lyons et al., 2016). Mutated genes that brought about boundary formation between a mutant and the parental strain were classified as kin discrimination (KD) loci. These are rather diverse and include contact‐dependent inhibition (CDI) (*wapAI*), and other microbial ‘attack and defence’ loci (*sdpABC, sdpIRs, skfA‐H*) that code for toxin and immunity proteins or peptide antibiotics (*sunA, bacA*). Additionally, among the KD loci are also regulators (*lytST, yvrHB, sigW*) that control response to antimicrobial attack or synthesis of antibiotics; histidine kinases (*ptkA* and *ptpZ*) that regulate synthesis of cell‐surface molecules; loci that modify cell wall structures (*lytC, dltA, tauD*), an operon that directs biosynthesis of an extracellular polysaccharide (*epsA‐O*) and the PhoR histidine kinase that regulates response to phosphate starvation (Lyons et al., 2016). Thus, kin discrimination in *B. subtilis* is a highly complex system that is influenced by multiple loci but the general conclusion is that these are in most cases directly or indirectly involved in the attack and defence strategies. Indeed, non‐kin strains like 168 (a domesticated version of NCIB 3610 (Earl et al., 2012) and RO‐NN‐1 (an isolate from the Mojave Desert (Cohan et al., 1991)) that show 97.97% average nucleotide identity, and very tight synteny have also many gaps of non‐conservation which could hide potential KD loci. Moreover, at the meeting point of two swarms one of the strains launches an attack, while the attacked one responds to damage by inducing a set of genes like SigW‐dependent stress response. However, the transcriptional response to attack is not uniform and it is again strain‐dependent (Lyons et al., 2016). Many questions remain unanswered regarding how the surface molecules contribute to kin discrimination, to what extent KD protects cooperative behaviours, how it shapes multicellular mode of microbial life and which evolutionary forces shape this social behaviour.

## Kin discrimination across the species barrier

It was recently revealed that *B. subtilis* kin discrimination is not restricted to members of the species but is extended to close relatives in the *B. subtilis* clade (Lyons and Kolter, 2017). As expected, representatives of more distant *B. cereus* clade and even less related strains were no longer subject to kin discrimination. This was tested using three assays; namely, a swarm meeting assay, the ability of two neighbouring colony biofilms to merge, and the detection of antibiosis halos that formed on lawn plates, where one strain was spotted onto a lawn of another (Lyons and Kolter, 2017). The strains used in this analysis were isolated by spore selection from soil samples from five locations and 38 were previously isolated strains from Genetic Stock Center and the American Culture Collection. Different assays were used to allow the strains to ‘meet’ under differing environmental conditions, each with their own timing implications. The authors found that for some strain combinations, the nature of interaction among isolates was influenced by the assay used: for example, pairs of isolates that formed kin interactions in one assay condition showed the opposite phenotype in another (Lyons and Kolter, 2017). These findings suggested that cooperation and antagonism can, in some cases, be context‐dependent. One hypothesis to explain the variable outcome of the interactions is that a context‐specific response is mediated by the factors involved in determining kin discrimination being made under different conditions. Therefore, bacteria could perhaps coexist in conditions under which both partners would benefit but compete when conditions would result in facultative cheating.

While strains and species in the immediate *B. subtilis* clade were almost always subject to kin discrimination, this was no longer the case after a specific relatedness ‘cut‐off point’. It was determined that species beyond the *B. pumilus* clade showed a random mixture of interactions against *B. subtilis* isolate NCIB 3610 and the KD phenotype have no longer correlated to relatedness (Fig. 5B). The loss of correlation is expected if some but not all of these more distant relatives inhabited different environmental niches, so that selection for or against kin discrimination has not occurred. However, further research is needed to explore the niche breath of the isolates and how niche traits shape KD. To test what the reason behind this cut‐off point for kin discrimination could be, an assay was developed for determining the ability of isolates to exploit each other’s public goods. Using a ‘surfactin stealing assay’ the authors found a correlation between the ability of an isolate to exploit the secreted products of another and antagonism (Lyons and Kolter, 2017). These findings strengthen the hypothesis that kin discrimination is involved in reducing social cheating.

## Division of labour in biofilm formation

Biofilm formation is arguably the most common multicellular behaviour exhibited by bacteria in nature (Stoodley et al., 2002). As mentioned earlier, biofilms consist of cells attached to each other or a surface and encased in an extracellular matrix produced by the biofilm members. In nature, many biofilms are comprised of multiple different species (Madsen et al., 2018). Due to the complexity of such systems, however, most of the current molecular knowledge on biofilm formation has been acquired using isogenic models. *B. subtilis* forms architecturally complex communities on agar surfaces (Branda et al., 2001), at the liquid to air interface of standing cultures (Branda et al., 2001), on the roots of plants (Beauregard et al., 2013) and on microtitre plate wells, submerged in buffer (Hamon and Lazazzera, 2001; Bridier et al., 2011). The process of biofilm formation in *B. subtilis* has primarily been studied under laboratory conditions with the undomesticated isolate NCIB 3610 (Branda et al., 2001). However, a naturally competent soil isolate of *B. subtilis*, PS‐216 (Stefanic and Mandic‐Mulec, 2009), which forms highly structured biofilms under laboratory conditions (Spacapan et al., 2018) and on plant roots (Stefanic et al., 2015) has been sequenced (Durrett et al., 2013) and also serves as an excellent model to study biofilms. NCIB 3610 shows limited competency due to the presence of the plasmid‐encoded (pBS32) protein ComI, which interferes with the competency machinery (Konkol et al., 2013). PS‐216 is naturally competent, and while there is no published genomic comparison between the two isolates, two explanations can be posited: first, in natural isolates variations in the level of phosphorylated DegU influences the degree of competence for transformation (Miras and Dubnau, 2016) and second, PS‐216 does not carry pBS32 (Durrett et al., 2013), and is therefore likely to lack the *comI *gene. In many cases, a buffered defined medium using glutamic acid, as the sole nitrogen source, and glycerol, as the sole carbon source, is used to trigger production of the biofilm matrix (Branda et al., 2001). A few studies have also examined biofilm formation of model strains in different growth conditions (Dogsa et al., 2013; Ma et al., 2017), while limited insights into the diversity of biofilm regulation and biofilm properties of different *B. subtilis* isolates have been acquired (Oslizlo et al., 2015; Sanchez‐Vizuete et al., 2015; Yu et al., 2016).

Through the use of single‐cell transcriptional fluorescent reporter fusions within a biofilm of the model isolate NCIB 3610 it has been revealed that, genetically identical cells differentiate into physiologically distinct cell types resulting in phenotypic heterogeneity (Chai et al., 2008; Vlamakis et al., 2008). Different cell types in the population are yielded that produce a range of public goods which can be shared between community members. Examples of public goods produced by biofilm members include exoproteases (Marlow et al., 2014), surfactants, such as surfactin (Branda et al., 2001) and the biofilm matrix components themselves (Chai et al., 2008). The macromolecules found in the extracellular matrix are crucial for biofilm development and in *B. subtilis* there are two major components of the matrix: the exopolysaccharides (Eps) (Branda et al., 2001) and protein fibres formed by TasA (Branda et al., 2006) that act synergistically with a bacterial hydrophobin called BslA (Ostrowski et al., 2011) which renders the community hydrophobic (Epstein et al., 2011; Kobayashi and Iwano, 2012). In addition, the matrix is rich in DNA and the ratios between polysaccharides, proteins and DNA depend on growth media composition (Dogsa et al., 2013). Knowledge about the division of labour during biofilm formation in *B. subtilis* began with the observation of phenotypic heterogeneity with regards to Eps and TasA production in an isogenic cell population (Chai et al., 2008). It was found that production of Eps is energetically expensive for individual cells and the Eps itself acts as a public good that benefits both the Eps producers and non‐producers alike (van Gestel et al., 2014). In spatially mixed populations, made up of a co‐culture of *eps *mutants and Eps producers, the *eps* mutants have a competitive advantage by exploiting the Eps produced by their neighbouring cells, without investing energy in its production. In spatially segregated communities, however, the *eps *mutants have a competitive disadvantage. The spatial segregation means the *eps* mutants are not surrounded by the Eps that is secreted by the wild type. The lack of the Eps makes the *eps* mutants unable to expand across the surface and they become outcompeted by the spreading wild‐type parental strain (van Gestel et al., 2014). Thus, spatial distribution provides another mechanism for reducing social cheating through exploitation of secreted public goods. Similar observations were recently reported for the second major component of the extracellular matrix, TasA. This fibrous protein, which is known to be produced by a subpopulation of the community was also shown to be costly for individual members to produce (although less so than Eps) and, similarly to Eps, albeit to a lesser extent, acts as a shared public good (Dragos et al., 2018).

## Quorum sensing during biofilm development

At the molecular level, division of labour in the isogenic population is a highly complex and tightly regulated process. The differentiation of genetically identical sister cells into phenotypically heterogeneous populations is called ‘bimodality’ and involves the regulators Spo0A (Fujita and Losick, 2005; Chai et al., 2008), DegU (Verhamme et al., 2007) and ComA (Nakano et al., 1991a; Stanley and Lazazzera, 2005). Each of these regulators is heavily influenced by quorum sensing, through both the ComQXPA and Rap‐Phr systems (Lopez and Kolter, 2010). In non‐matrix producing cells, transcription of the matrix operons (namely *tapA‐sipW‐tasA* and *epsA‐O*) is repressed when SinR binds to the promoters (Kearns et al., 2005; Chu et al., 2006). SinR is part of a double negative feedback loop with another regulator called SlrR, such that SinR represses *slrR *transcription (Chai et al., 2010). The presence of intermediate levels of phosphorylated Spo0A triggers production of SinI (Shafikhani et al., 2002; Fujita et al., 2005), which is an antagonist of SinR (Bai et al., 1993). SinI binds to SinR and represses its action, thus allowing the matrix operons and *slrR *to be expressed (Kearns et al., 2005; Chu et al., 2006; Chu et al., 2008). Once SlrR is produced, it binds to SinR and prevents SinR from inhibiting transcription of its own coding region (Chai et al., 2010). The SlrR‐SinR complex also acts to prevent the expression of genes involved in cell separation and motility, resulting in differentiation of cells into non‐motile biofilm matrix producers (Chai et al., 2010). This is an epigenetic switch that is stable across multiple generations (Norman et al., 2013).

The epigenetic switch that differentiates motile cells into matrix producers provides an example for the influence that quorum sensing has on cell differentiation and biofilm formation. Although matrix gene expression is not directly controlled by a quorum sensing system, ComA is indirectly involved in matrix production. As detailed earlier, ComA is the response regulator of the ComXQPA QS system, and controls surfactin production and genetic competence (Magnuson et al., 1994). Production and secretion of surfactin in the extracellular environment causes potassium leakage in neighbouring cells (Lopez et al., 2009a). Potassium leakage triggers phosphorylation and activation of Spo0A which, when at intermediate levels in the cell, de‐represses the biofilm matrix operons, as discussed previously, leading to the production of the biofilm matrix that encases the community (Lopez et al., 2009a; Lopez et al., 2009c). Other extracellular products present in the biofilm which are controlled by QS include exoproteases. In contrast to ‘weaker’ biofilm forming ‘domesticated’ laboratory strains (such as JH642, 168 and PY79), wild or undomesticated isolates of *B. subtilis*, including the model strain NCIB 3610 and the soil isolate PS‐216 form structured biofilms under laboratory conditions. Interestingly, one of the main differences between domesticated and undomesticated strains is in the production of DegQ, which connects the ComQXPA system with DegU (Stanley and Lazazzera, 2005; McLoon et al., 2011; Miras and Dubnau, 2016; Spacapan et al., 2018), ultimately leading to expression of extracellular proteases. As a result, wild isolates which can produce DegQ, show higher levels of exoprotease and secondary metabolite expression. It has been experimentally demonstrated that deletion of *comQ*, essential for the production of ComX, the signal peptide of the ComQXPA system, results in strong reduction of exoprotease expression in static biofilm cultures of PS‐216 (Spacapan et al., 2018). Although it could be speculated that high proteolytic activity in biofilms may promote biofilm dispersal, recent experiments show that the TasA fibres present in the matrix are highly resistant to proteolytic degradation (Erskine et al., 2018). Interestingly however, signalling peptides such as ComX, are sensitive to exoprotease degradation (Spacapan et al., 2018), adding to the complexity of the regulation of public good production in bacterial communities. It could be that proteolysis is a general QS quenching mechanism that may have important implication for the dynamics of peptide‐based signalling in *B. subtilis* and relatives. For example ComX is widespread in Firmicutes (Dogsa et al., 2014), with many Gram‐positive bacteria applying signalling peptides as canonical QS signals (Kleerebezem et al., 1997) and the majority of quorum sensing systems regulating synthesis of extracellular proteases (Hense and Schuster, 2015). It will be of interest to identify if there is species‐specificity in the degradation of ComX, or if promiscuous proteolytic activity occurs. This would impact the stability of signalling systems and have implications for how single‐species and mixed‐species communities develop.

In addition to ComXQPA, the Rap‐Phr systems also influence the activity of the master regulators involved in cell differentiation, in concert with ComA, Spo0A (through Spo0F) and DegU, as described in previous sections. For example, the RapP‐PhrP quorum sensing cassette, encoded on a large plasmid in NCIB 3610, has been found to play a role in controlling biofilm architecture (Omer Bendori et al., 2015). Collectively, these regulators control the expression of multiple genomic regions to regulate processes such as production of the extracellular matrix, exoproteases, development of genetic competence and as a last survival strategy sporulation (Lopez et al., 2009b, Lopez and Kolter, 2010), contributing to the survival of the biofilm members under diverse environmental conditions.

## Long‐range metabolic signalling in the biofilm

**Figure 6:**
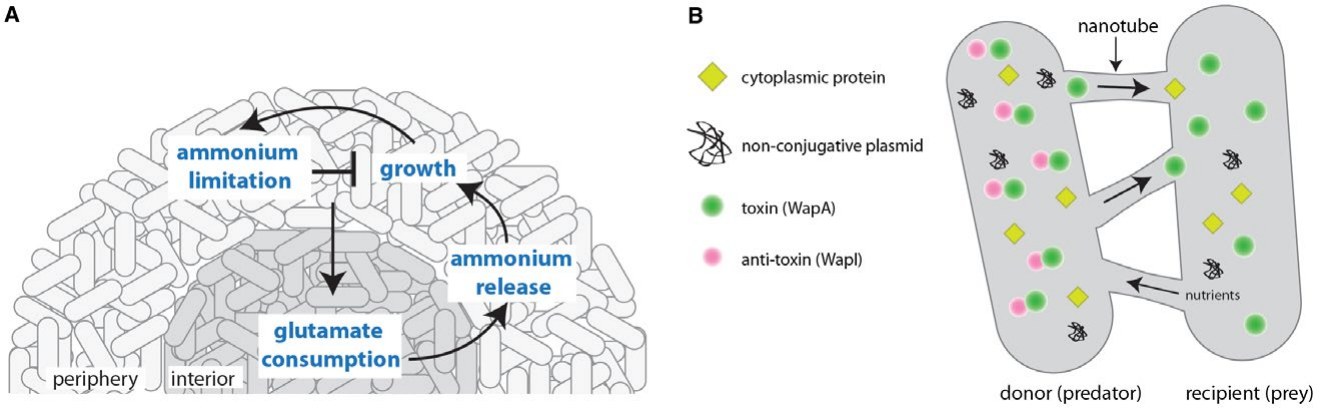
Long‐range and contact‐dependent communication in *B. subtilis*. A. Long‐range metabolic signalling occurs in developing biofilm communities and results in oscillations between growth and growth inhibition. B. Contact‐dependent communication between *B. subtilis* and other cells (either *B. subtilis* or other species) occurs using nanotubes. Cytoplasmic contents can be moved from donor to recipient cells, while small nutrient molecules can be extracted from the prey cell by the predator, demonstrating bidirectional movement of molecules.

Intimate cooperation within the biofilm community, mediated by the control of gene regulation, is coupled with an intrinsic need for the resident bacteria to compete or cooperate to access available nutrients. In addition microscopy‐based analysis of two‐dimensional biofilms formed by NCIB 3610, in a constant nutrient environment, has revealed that when the communities exceed a certain size (an average diameter of 580 ± 85 μm) they exhibit periodic arrests of growth (oscillations) (Liu et al., 2015). These collective oscillations were sustained for more than one day, where the average periodicity was 2.5 ± 0.8 h. The oscillations in growth were found to be controlled by a long‐range metabolic co‐dependence between the cells in the periphery of the biofilm with those in the interior regions (Fig. 6A). The cessation of cell growth at the biofilm periphery is linked with ammonium limitation and this transient growth arrest allows cells in the interior of the biofilm to acquire, and consume, glutamate from the medium. The cells generate ammonium that is accessed by the cells in the periphery, thus allowing them to consume glutamate, restoring growth again, albeit transiently. The periodic ammonium starvation at the biofilm periphery occurs as ammonium, produced in this zone during glutamate utilisation, and is released into the extracellular medium, thereby becoming inaccessible to the cells (Liu et al., 2015). The overall bacterial population was found to benefit from the process of oscillating periods of biofilm growth and arrest, with an increase in the overall level of cell survival after exposure to extracellular stress (Liu et al., 2015). Therefore, while the cells in the biofilm periphery transiently starve those in the interior, they also protect them (Liu et al., 2015).

The metabolic coordination in the two‐dimensional *B. subtilis* biofilm was later found to depend on electrochemical signalling (Prindle et al., 2015). Metabolically starved cells located in the interior of the biofilm collectively trigger depolarisation of the cell membranes of those situated at the biofilm periphery, through a sudden release of potassium. This limits the ability of cells to take up glutamate, to retain ammonium, and thus to grow, allowing cells in the interior access to the nutrients (Prindle et al., 2015). The coordination of metabolic activity is not restricted to within one biofilm. When *B. subtilis* biofilms are found in close, but non‐touching proximity, synchronised oscillations in growth develop in the two physically separate communities (Liu et al., 2017). The reach of the electrical signal goes beyond communication within biofilms as potassium release from a mature oscillating biofilm can stimulate recruitment of motile cells to biofilm edge where they get subsumed into the developing structure (Humphries et al., 2017). The ability to recruit motile cells to the oscillating biofilm bypasses the species barrier with motile *Pseudomonas aeruginosa* cells being attracted by the electrical signal released by the *B. subtilis* biofilm (Humphries et al., 2017). These findings demonstrate the broad impacts of the long‐range signalling processes both within *B. subtilis *simple and mixed communities. This example of cooperation within an isogenic community to access and maximise nutrient sources is however not unique. In planktonically growing *B. subtilis *the population divides into two metabolically distinct, but dynamic, subpopulations: one which produces acetate and one that produces acetoin (Rosenthal et al., 2018). Therefore, it will be important to address how these intricate interactions manifest and impact communities in the natural environment where nutrients may exist in micro‐niches, biofilms will be more complex structurally and where multiple species will be present.

## Short‐range contact‐dependent communication

Intercellular communication between *B. subtilis* cells can be contact independent, as in the case of electrical signalling and quorum sensing, but also contact‐dependant. *B. subtilis *can directly exchange cytoplasmic molecules through (or using) tube‐like membranous appendages dubbed ‘nanotubes’ (Dubey and Ben‐Yehuda, 2011). Molecules that have been experimentally demonstrated to transfer between cells using the nanotubes have been heterologous ‘marker’ proteins which include the green fluorescent protein, calcein and antibiotic resistance proteins. For example, co‐culturing cells encoding antibiotic resistance cassettes with unmarked wild‐type cells resulted in transient antibiotic resistance of the wild‐type strain (Dubey and Ben‐Yehuda, 2011). The cells were also found to be able to transport DNA to their wild‐type neighbours using nanotubes, this was in the form of non‐conjugative plasmids that harboured antibiotic resistance cassettes, which resulted in the heritable resistance of the recipient cells (Dubey and Ben‐Yehuda, 2011) (Fig. 6B).

Research into the composition of these structures revealed that the protein YmdB is required for nanotube formation, and thus intercellular exchange of molecules (Dubey et al., 2016). Consistent with a role in structuring the *B. subtilis *community, YmdB was first identified as required for both biofilm formation (Diethmaier et al., 2011) and later for wild‐type colony formation on solid media (Mamou et al., 2016). YmdB is a phosphodiesterase with activity against 2′,3′‐ and 3′,5′‐cyclic nucleotide monophosphates that is required for the differentiation of motile‐cells into biofilm matrix producers, such that the absence of YmdB cause loss of gene expression bimodality, resulting in a population made up exclusively of short motile cells (Diethmaier et al., 2014). While there is a link between nanotube and biofilm formation, the extracellular biofilm matrix components themselves are not required for development of nanotubes, as *tasA *mutant strains are capable of forming functional structures (Dubey et al., 2016). Therefore, the function that nanotubes, and the consequential exchange of cytoplasmic contents, play in the formation of biofilms, if any, remains to be elucidated.

Nanotube formation, and the exchange of cytoplasmic contents, is not restricted to members of the same species. It has been shown that *B. subtilis *can transport molecules, through nanotubes, to both *Escherichia coli *and *Staphylococcus aureus *(Dubey et al., 2016). This means that nanotubes can play a role in interspecies competition. For example, *B. subtilis *can use nanotubes to transfer the toxic protein WapA into neighbouring *B. megaterium *cells, resulting in growth inhibition (Stempler et al., 2017). WapA is not toxic in *B. subtilis* strains that carry an anti‐toxin, WapI (Koskiniemi et al., 2013; Lyons et al., 2016). The nanotubes are capable of bidirectional movement of molecules as they allow *B. subtilis* to extract nutrients from rival *B. megaterium *cells (Stempler et al., 2017) (Fig. 6B). The fact that intimate connections are able to form between *B. subtilis* and both Gram‐positive and Gram‐negative species suggests a non‐specific interaction between the nanotube and the recipient cell. How the nanotube connects to a neighbouring cells through the thick cell wall of Gram‐positive bacteria or how the nanotube connects to and extends across the outer membrane of *E. coli* allowing passage of molecules into the cytoplasm remains unanswered. Nonetheless, nanotubes may potentially have an immense influence in the social life of *B. subtilis *in nature, contributing to both cooperative and antagonistic interactions.

## Concluding Remarks

The molecular basis of multicellular processes has been primarily studied in single‐genotype populations under laboratory conditions. However, this is, of course, not representative of the complexity and diversity which exists in nature. For example, the properties and functions of biofilms are greatly dependent on interactions between species and have been termed ‘community‐intrinsic properties’ (Madsen et al., 2018). Indeed a combination of four species in a biofilm was found to result in a 3‐4 times increase in the biomass compared with the single isolate biofilms of its constituent species. In this experiment, the number of cells belonging to each of the four species was all increased by comparison to growth in pure culture. Additionally, the spatial organisation of the members in the four‐species biofilm was unpredictable based on analysis of two species models (Burmolle et al., 2006). This demonstrates the immense influence that each species has on the community in terms of growth and structure. While the effect that diverse species have on biofilm formation in *B. subtilis* remain largely underexplored, other soil bacteria have been found to induce or repress *B. subtilis *biofilm formation (Powers et al., 2015). Repression of biofilm development has been described as a result of co‐culture of *B. subtilis* with soil isolates of *Pseudomonas putida *and *Pseudomonas protogens*. *P. protogens *was found to produce the antifungal 2,4‐diacetylphloroglucinol (DAPG), responsible for *B. subtilis *biofilm inhibition (Powers et al., 2015). In contrast, most of the soil species that could induce biofilm formation in *B. subtilis* were members of the genus *Bacillus *(Shank et al., 2011). The identity of the secreted molecules produced by these soil isolates is largely unknown but they induce biofilm matrix production through two mechanisms; (1) induction of matrix gene expression *via *the Spo0A~P pathway that is activated by the sensor kinase KinD or (2) by preferentially killing the non‐matrix‐producing cells in the population.

In addition to the direct effect that microorganisms have on each other in multicellular contexts, environmental conditions are also critical to shaping social interactions among microbes. As discussed above, this was demonstrated in *B. subtilis*, where growth under different multicellular conditions influenced the nature of the interactions among isolates (Lyons and Kolter, 2017). This is not specific to *Bacillus *species as similar findings have also been shown for *Pseudomonas aeruginosa* and *Staphylococcus aureus* which usually do not coexist, as *P. aeruginosa* outcompetes *S. aureus* through production of molecules that are under the control of QS systems. In the blood however, QS signalling is inhibited in *P. aeruginosa* due to binding on serum albumin to QS molecules, resulting in coexistence of the two organisms (Smith et al., 2017). Therefore, it will be interesting to address the relationship between kin discrimination, quorum sensing and cheating in the formation, competitive fitness and spatial organisation of cells within in environmental biofilms and couple this with an analysis of the impact exerted by diverse environmental settings.

## Declarations of interest

None.
